# Inclusive Leadership Is Associated With Nurse‐Perceived Nurse‐Patient Relationship Quality Through the Mediation Link of Self‐Control and Resilience: A Cross‐Sectional Study

**DOI:** 10.1155/jonm/7165226

**Published:** 2026-06-26

**Authors:** Yue Wen, Linli Zhuang, Song Wang

**Affiliations:** ^1^ Department of Gastrointestinal Surgery, West China Hospital, Sichuan University, Chengdu, China, scu.edu.cn; ^2^ West China School of Nursing, Sichuan University, Chengdu, China, scu.edu.cn; ^3^ Department of Rheumatology and Immunology, West China Hospital, Sichuan University, Chengdu, China, scu.edu.cn; ^4^ Department of Radiology, West China Hospital, Sichuan University, Chengdu, China, scu.edu.cn

**Keywords:** inclusive leadership, mediation, nurse–patient relationship, resilience, self-control

## Abstract

**Background:**

The nurse–patient relationship is a cornerstone of high‐quality care, yet its cultivation is challenged by the demanding clinical environment. Although inclusive leadership is recognized as a supportive organizational factor, the precise psychological mechanisms through which it is linked to this relationship remain underexplored. This study examines whether self‐control and resilience mediate the link between inclusive leadership and nurse‐perceived nurse–patient relationship quality, both independently and in sequence.

**Methods:**

A cross‐sectional survey of 429 registered nurses working in hospitals in China’s Sichuan Province was conducted in accordance with the STROBE guidelines. Participants completed validated scales to measure their perceptions of inclusive leadership, self‐control, resilience, and the nurse–patient relationship. Data were analyzed using descriptive statistics, confirmatory factor analysis, correlation analysis, and mediation analysis with the Hayes PROCESS macro (Model 6).

**Results:**

There were positive correlations between inclusive leadership and self‐control, resilience, and the nurse‐perceived nurse–patient relationship (rs > 0.27, ps < 0.001). Mediation analysis confirmed a significant direct link between inclusive leadership and the nurse‐perceived nurse–patient relationship (*β* = 0.18, 95% CI: [0.08, 0.28]). The total indirect effect was also significant (*β* = 0.25, 95% CI: [0.18, 0.32]). Self‐control (*β* = 0.05, 95% CI: [0.02, 0.07]) and resilience (*β* = 0.18, 95% CI: [0.12, 0.25]) mediated the relationship independently. Furthermore, a theoretically specified sequential indirect pathway through self‐control and resilience was statistically supported (*β* = 0.02, 95% CI: [0.01, 0.04]).

**Conclusion:**

The findings provide initial evidence suggesting that inclusive leadership is associated with a stronger nurse‐perceived nurse–patient relationship, with this association being mediated by self‐control and resilience, both independently and sequentially. Healthcare organizations should consider developing inclusive leadership and implementing interventions that concurrently strengthen nurses’ self‐regulatory capacity and resilience to cultivate optimal therapeutic relationships.

## 1. Introduction

Nursing practice is fundamentally relational, with the quality of the nurse–patient relationship serving as a cornerstone of therapeutic care, patient satisfaction, and clinical outcomes [1]. This relationship is a dynamic therapeutic alliance that directly influences patient adherence, health outcomes, and perceptions of care quality [2]. In complex, high‐stress clinical settings, fostering robust, trusting, and patient‐centered relationships is a crucial indicator of high‐quality nursing practice [3]. However, maintaining these optimal relationships is continually challenged by systemic pressures, high patient acuity, emotional labor, and the complex psychological demands placed on nursing staff. Consequently, identifying modifiable organizational factors associated with nurses’ ability to cultivate and maintain positive patient relationships is a critical imperative for healthcare management.

Leadership style has been consistently identified as a pivotal upstream factor shaping the practice environment, influencing team dynamics, staff well‐being, and care delivery [4]. While transformational and authentic leadership models have shown promise, recent focus has shifted toward a more relational and facilitative paradigm: inclusive leadership [5]. Conceptualized by Carmeli and colleagues, inclusive leadership is characterized by a leader’s intentional openness (soliciting and valuing followers’ opinions), accessibility (being approachable for communication), and availability (providing time and support) [6]. This leadership approach is associated with a climate of psychological safety—where individuals feel valued for their uniqueness and experience a sense of belonging—which is conducive to both nurse satisfaction and enhanced professional conduct [7]. While transformational leadership focuses on inspiration and idealized influence, and authentic leadership emphasizes self‐awareness and transparency, inclusive leadership specifically centers on the leader’s intentional openness, accessibility, and availability to followers [4, 5]. In the hierarchical and often stressful context of nursing, inclusive leadership may counteract feelings of powerlessness and voicelessness, potentially freeing cognitive and emotional resources that nurses can then direct toward patient care [8]. A growing body of empirical research has revealed that inclusive leadership is positively linked with job outcomes. For example, it has been shown to improve nurses’ work engagement and innovation [9, 10], while also reducing absenteeism, job burnout, and intention to leave [11–13]. However, the specific pathways through which a nurse’s perception of an inclusive leader is linked to more effective, trusting, and patient‐centered interactions at the bedside remain inadequately mapped. The link is likely not direct but filtered through the nurse’s internal psychological resources. The present study posits that two critical, trainable resources—self‐control and resilience—serve as key explanatory mechanisms, operating both independently and in sequence to bridge leadership behavior and relational outcomes.

The first proposed pathway centers on self‐control, defined as the capacity to override impulsive reactions, regulate emotions, and persist in goal‐directed behavior despite temptations or fatigue [14]. This capacity is paramount in nursing, where professionals must consistently manage their own emotional and physiological responses to provide calm, consistent, and professional care [15]. Drawing from the conservation of resources (COR) theory [16], an inclusive leader acts as a key social resource that may reduce unnecessary resource drains. A leader who is open, accessible, and available may minimize role ambiguity, reduce supervisory‐based stress, and provide instrumental support. This supportive environment may conserve the nurse’s finite self‐regulatory resources that would otherwise be depleted by navigating a fraught or unsupportive supervisory relationship [17]. With these resources preserved, nurses may have a greater reservoir of volitional capacity to deploy in clinical encounters [18]. This effective self‐regulation may facilitate the building of trust and rapport [19], as patients experience the nurse as predictable, professional, and attentive—core components of a positive therapeutic relationship.

The second independent pathway may operate through resilience, the adaptive process of weathering adversity, managing stress, and maintaining or regaining psychological well‐being and functional competence [20]. While self‐control manages moment‐to‐moment impulses, resilience provides the broader, sustained capacity to endure and grow from cumulative workplace challenges. Inclusive leadership is theorized to be a potent catalyst for resilience development. Through the mechanisms outlined in social cognitive theory [21], inclusive leaders may provide mastery experiences (by empowering nurses and trusting their judgment), vicarious learning (by modeling adaptive coping), and social persuasion (through affirming feedback and encouragement) [22]. When nurses feel their contributions are valued and that they have a secure base of leader support, their belief in their own ability to cope with and recover from difficulties is fortified [23]. This enhanced resilience may translate directly to the nurse–patient relationship. A resilient nurse is less likely to succumb to compassion fatigue or cynicism, thereby preserving the empathy essential for patient‐centered care [24, 25]. Consequently, resilience may enable the sustained emotional investment and positive outlook necessary for building and maintaining strong therapeutic alliances over time.

Beyond their independent roles, we propose a sequential pathway where self‐control and resilience function in a chain linking inclusive leadership with the nurse‐perceived nurse–patient relationship. This proposition is grounded in the COR theory principle of “resource caravans” [26], whereby resources aggregate and foster the development of other resources. In this chain, self‐control acts as a foundational, stabilizing resource. The successful daily exercise of self‐control—managing irritations, adhering to protocols under pressure, and regulating emotional responses—may prevent the chronic, minor resource losses that can contribute to a state of depletion known as burnout [18, 27]. By consistently conserving and effectively managing personal resources, a nurse may maintain a stable psychological platform. This platform of regulatory stability may be critical for the development and maintenance of resilience [20]. A nurse who is not perpetually in a state of regulatory exhaustion may be more cognitively and emotionally available to engage in the positive reappraisal, solution‐focused coping, and seeking of support that characterize resilient functioning. Previous studies have established that self‐control acts as an antecedent to psychological resilience in different populations [28–30]. However, it is important to acknowledge that alternative directional relationships (e.g., resilience preceding self‐control) are theoretically possible. The proposed ordering is a hypothesis‐driven model and should not be interpreted as evidence that self‐control precedes resilience in time. This sequential model illustrates a dynamic psychological process whereby leadership support may initiate a positive cascade: first facilitating better daily self‐regulation, which in turn may sustain and amplify long‐term adaptive capacity, with the compounded benefit being a superior ability to connect with and care for patients.

### 1.1. The Present Study

While inclusive leadership, self‐control, resilience, and the nurse–patient relationship have been studied in various pairings, their integration into a comprehensive model remains absent. Addressing this gap is essential for moving from descriptive correlation to a mechanistic understanding that can inform precise interventions. Therefore, guided by COR theory [16, 26], this study aims to investigate a sequential mediation model to elucidate the psychological bridge between leadership and relational care. We hypothesize that: (1) Inclusive leadership will be positively associated with the quality of the nurse‐perceived nurse–patient relationship. (2) Self‐control will independently mediate the association between inclusive leadership and the nurse‐perceived nurse–patient relationship quality. (3) Resilience will independently mediate the association between inclusive leadership and the nurse‐perceived nurse–patient relationship quality. (4) Self‐control and resilience will sequentially mediate this association, such that inclusive leadership is associated with greater self‐control, which is subsequently linked with higher resilience, ultimately linking to an enhanced nurse‐perceived nurse–patient relationship quality.

## 2. Methods

### 2.1. Research Design

This study employed a cross‐sectional, quantitative survey design to investigate the relationships between inclusive leadership, self‐control, resilience, and the nurse‐perceived nurse–patient relationship quality. The primary aim was to test a sequential mediation model proposing that self‐control and resilience—both individually and in series—link inclusive leadership and nurse‐perceived nurse–patient relationship quality. The study was conducted and reported in accordance with the Strengthening the Reporting of Observational Studies in Epidemiology (STROBE) guidelines for cross‐sectional studies [31].

### 2.2. Ethical Standards

The study protocol received formal approval from the Ethics Committee of West China Hospital of Sichuan University (Approval No.: 2021‐1216). Prior to participation, all potential respondents were provided with a detailed information sheet outlining the study’s purpose, procedures, potential benefits, and assurances of confidentiality. Participation was entirely voluntary, and written informed consent was obtained from each nurse. To protect participant anonymity, all collected data were de‐identified, and no personal identifiers were stored with the survey responses.

### 2.3. Participants and Procedure

A convenience sampling method was used to recruit registered nurses from 2 secondary and 2 tertiary public hospitals in Sichuan Province, China. Hospitals were purposively selected based on existing research collaborations and willingness to participate, aiming to ensure a diverse range of institutions. Data collection took place over a 2‐month period (July–August) in 2025 via an online questionnaire survey platform (https://www.wjx.cn/) distributed by trained research assistants within the participating hospitals. Participation was voluntary and anonymous; nurses were explicitly assured that their responses would not be shared with their supervisors to minimize social desirability bias. The inclusion criteria were (1) holding a valid registered nurse license in China, (2) having at least 1 year of full‐time clinical nursing experience, and (3) providing informed consent. Nursing students and nurses in provisional training programs were excluded. Of the 448 nurses initially invited, 429 completed the survey with valid responses, resulting in a response rate of 95.8%. Nineteen questionnaires were excluded due to substantial missing data, patterned responses (e.g., straight‐lining), or signs of inattention. Due to concerns for participant anonymity, departmental affiliation was not systematically recorded. While we acknowledge that departmental context (e.g., unit acuity, patient flow, and team composition) can influence study variables, we were unable to collect this information. This was to prevent potential identification of participants, particularly in smaller departments like intensive care units or emergency departments where nurse numbers are limited.

### 2.4. Research Measures

#### 2.4.1. Inclusive Leadership Scale (ILS)

Participants’ perceptions of inclusive leadership were assessed using the ILS [6]. One example item was ‘My nurse manager is open to hearing new ideas’. The ILS contains nine items structured across three core dimensions: openness, accessibility, and availability. Participants rated their level of agreement with each statement on a five‐point Likert scale (1 = “strongly disagree” to 5 = “strongly agree”). An aggregated score ranging from 9 to 45 was calculated by summing all item responses, with higher scores reflecting a greater perceived level of inclusive leadership. The ILS has demonstrated acceptable psychometric properties, including reliability and validity, specifically within Chinese nursing samples [10, 13]. In this investigation, the scale exhibited strong internal consistency (Cronbach’s *α* = 0.92).

#### 2.4.2. Brief Self‐Control Scale (BSCS)

The capacity for self‐control was measured using the BSCS [32]. This tool is structured as a unidimensional instrument comprising 13 items. Respondents indicated their level of agreement using a five‐point Likert scale, where 1 signified “strongly disagree” and 5 signified “strongly agree.” After reverse‐scoring several items (2, 3, 4, 5, 7, 9, 10, 12, and 13), the total self‐control score was then computed by summing all item responses, with higher scores indicating higher levels of perceived self‐control. The SCS is well‐validated and has proven effective in Chinese settings, reliably measuring self‐control, particularly in nursing populations [33, 34]. In the present investigation, the scale achieved adequate internal consistency, yielding a Cronbach’s *α* of 0.82.

#### 2.4.3. 10‐Item Connor‐Davidson Resilience Scale (CD‐RISC‐10)

Participants’ resilience levels were evaluated using the CD‐RISC‐10 [35]. This instrument is structured as a unidimensional measure where participants rated their agreement with statements on a five‐point Likert scale (1 = “strongly disagree” to 5 = “strongly agree”). A total score was calculated by summing all item responses; consequently, elevated scores signified a higher degree of resilience. The Chinese version of the CD‐RISC‐10 has been validated for use across diverse groups, including nurses, showing satisfactory psychometric properties [36, 37]. The scale showed excellent internal consistency in this study, with Cronbach’s *α* of 0.95.

#### 2.4.4. Nurse–Patient Relationship Scale (NPRS)

The relationship between nurses and patients was evaluated via the NPRS developed by Ma et al., utilizing the nurses’ viewpoint in a Chinese context [38]. It is important to note that this measure captures nurses’ perceptions of the relationship rather than patient‐reported experiences. This instrument measures two underlying constructs: nurse–patient trust and patient‐centered care. Each item was rated using a six‐point Likert format, spanning from 1 (“strongly disagree”) to 6 (“strongly agree”). Crucially, higher resulting total scores on this scale signify an improvement or strength in the nurse–patient relationship. The scale has been shown to be reliable and valid for measuring this relationship in different nursing populations [39, 40]. In the present investigation, the measure achieved a high degree of internal consistency, as indicated by a Cronbach’s *α* of 0.96.

#### 2.4.5. Demographic and Professional Measures

Participants were asked to report their age, sex, annual income, years of formal education, length of nursing work experience, educational level, marital status, and professional title. These measures were collected for descriptive purposes and included as covariates in adjusted analyses.

### 2.5. Data Analyses

Data were analyzed using IBM SPSS Statistics (Version 26.0) and the PROCESS macro (Version 4.1, Model 6) developed by Hayes [41]. First, a Harman’s single‐factor test was performed to investigate potential common method bias (CMB) in the self‐report measures [42]. The procedure required an exploratory factor analysis of all measurement items. According to established guidelines [42, 43], evidence of significant CMB is deemed absent if a single factor fails to account for more than 40% of the total variance. However, it should be noted that Harman’s test is a conservative diagnostic, and the absence of a dominant factor reduces but does not eliminate concern for CMB. Second, descriptive statistics (mean, standard deviation [SD], and frequency) were calculated for all demographic and study variables. The normality of the distribution for key study variables was assessed using skewness and kurtosis statistics (all values ranging between −1 and +1) [44]. The assumptions of linearity (assessed via scatterplots), homoscedasticity (assessed via residual plots), and absence of multicollinearity (all variance inflation factor [VIF] values < 2) were tested and met. Then, bivariate Pearson correlations were computed to examine the preliminary relationships among inclusive leadership, self‐control, resilience, and nurse‐perceived nurse–patient relationship quality.

To assess the construct validity in the measurement model, a confirmatory factor analysis (CFA) was conducted using AMOS software (Version 22.0) [45]. The hypothesized four‐factor model (inclusive leadership, self‐control, resilience, and nurse‐perceived nurse–patient relationship quality) was tested against alternative models (e.g., three‐factor and one‐factor models). Model fit was evaluated using the comparative fit index (CFI), Tucker–Lewis index (TLI), root mean square error of approximation (RMSEA), and standardized root mean square residual (SRMR). Discriminant validity was assessed using the Heterotrait–Monotrait (HTMT) ratio of correlations, with values below 0.85 indicating adequate discriminant validity [46].

The core analysis tested the hypothesized sequential mediation model. Using the PROCESS Model 6 [41], we examined (a) the direct effect of inclusive leadership (X) on the nurse‐perceived nurse–patient relationship quality (Y) and (b) the indirect effects through self‐control (M1) and resilience (M2) as parallel and serial mediators. The significance of the indirect effects was determined using bias‐corrected bootstrapping with 5000 resamples. Effects were considered statistically significant if the 95% confidence interval (CI) did not include zero. This analytical approach was also applied separately to the two subdimensions of the outcome variable—nurse–patient trust and patient‐centered nursing—as detailed in the Supporting Information. Notably, all variables were standardized before the PROCESS modeling analyses. The bootstrap indirect effects were standardized, and sociodemographic variables were included as the covariates in every equation of the PROCESS model. Specifically, sex was included because previous evidence shows that female nurses may have different expectations and sensitivities regarding leader accessibility than male nurses [47]. Age and years of experience were included because more experienced nurses may develop greater self‐regulatory capacity [48]. Educational level and income were included as proxies for socioeconomic status, which may influence workplace perceptions [49].

## 3. Results

### 3.1. Participant Characteristics

The sociodemographic and professional characteristics of the 429 participating nurses are summarized in Table 1. The sample was predominantly female (93.7%, *n* = 402). Participants had a mean age of 34.46 years (SD = 8.14, range: 19–60) and an average of 12.74 years of nursing experience (SD = 8.62). The majorities held a bachelor’s degree (78.3%) and were married (67.8%). The average annual income was 84,500 CNY (SD = 36,900). Professional titles were distributed across primary (42.6%), intermediate (47.1%), and vice‐senior/senior levels (8.4%).

**TABLE 1:** Sociodemographic characteristics of the participants.

Variable	Mean ± SD (range) *N*%
Sex	
Male	27 (6.3%)
Female	402 (93.7%)
Age (years)	34.46 ± 8.14 (19–60)
Income (CNY/year)	84500 ± 36900 (20000–260000)
Years of education	15.80 ± 0.64 (12–19)
Length of nursing work (years)	12.74 ± 8.62 (1–41)
Educational level	
Secondary vocational degree	4 (0.9%)
College degree	84 (19.6%)
Bachelor degree	336 (78.3%)
Graduate degree	5 (1.2%)
Marital status	
Married	291 (67.8%)
Unmarried	118 (27.5%)
Divorced	15 (3.5%)
Widowed	3 (0.7%)
Other	2 (0.5%)
Professional title	
None	8 (1.9%)
Primary	183 (42.6%)
Intermediate	202 (47.1%)
Vice senior	32 (7.5%)
Senior	4 (0.9%)

*Note:* N, number.

Abbreviation: SD, standard deviation.

### 3.2. CMB Examination

The exploratory factor analysis demonstrated that the shared variance among the study measures was dispersed across several dimensions, rather than being concentrated in a dominant factor. We identified six factors exhibiting eigenvalues greater than one. Critically, the variance explained by the primary factor was modest, accounting for only 25.58% of the total variance—a value below the commonly employed 40% benchmark for strong common factor dominance. This distribution pattern suggests that significant CMB is less likely to be a primary concern, although the single‐source, self‐report nature of the data means that some inflation of associations cannot be ruled out.

### 3.3. CFA and Construct Validity

The hypothesized four‐factor model demonstrated good fit with the data: *χ* ^2^ = 1847.56, *p* < 0.001; CFI = 0.94, TLI = 0.93, RMSEA = 0.06, SRMR = 0.05. All factor loadings were significant (*p* < 0.001), supporting convergent validity. This four‐factor model fit significantly better than alternative models, including a three‐factor model (combining self‐control and resilience: Δ*χ* ^2^ = 156.34, *p* < 0.001) and a one‐factor model (Δ*χ* ^2^ = 892.45, *p* < 0.001). Discriminant validity was supported as all HTMT ratios were below the 0.85 threshold (HTMT values ranged from 0.41 to 0.68).

### 3.4. Descriptive Statistics and Bivariate Correlations

The means, SDs, and intercorrelations for the study variables are presented in Table 2. All variables demonstrated acceptable levels of skewness and kurtosis (values between −1 and +1), indicating normal univariate distributions [44]. As hypothesized, inclusive leadership was positively correlated with self‐control (*r* = 0.27, 95% CI: [0.18, 0.36]), resilience (*r* = 0.57, 95% CI: [0.49, 0.63]), and the overall nurse‐perceived nurse–patient relationship quality (*r* = 0.41, 95% CI: [0.32, 0.51]). Both self‐control (*r* = 0.36, 95% CI: [0.28, 0.44]) and resilience (*r* = 0.53, 95% CI: [0.45, 0.59]) were also positively correlated with nurse‐perceived nurse–patient relationship quality. The two subscales of the outcome, nurse–patient trust and patient‐centered nursing, showed similar correlation patterns with the predictor and mediators. Furthermore, multicollinearity diagnostics indicated no concerns (all VIF values < 2).

**TABLE 2:** Descriptive statistics and bivariate correlations of study measures.

Measure	Mean ± SD	Skewness	Kurtosis	1	2	3	4	5
1. ILS	38.98 ± 6.00	−0.78	0.55	—				
2. BSCS	44.72 ± 8.11	0.15	−0.54	0.27	—			
3. CD‐RISC‐10	41.09 ± 6.40	−0.28	−0.29	0.57	0.37	—		
4. NPRS	48.51 ± 5.85	−0.88	0.08	0.41	0.36	0.53	—	
5. NPRS_NPT	21.24 ± 2.82	−0.75	−0.05	0.39	0.34	0.52	0.96	—
6. NPRS_PCN	27.28 ± 3.25	−0.99	0.24	0.41	0.35	0.49	0.97	0.86

*Note:* All correlation coefficients were statistically significant at the *p* < 0.001 level.

Abbreviations: BSCS, Brief Self‐Control Scale; CD‐RISC‐10, 10‐Item Connor‐Davidson Resilience Scale; ILS, Inclusive Leadership Scale; NPRS, Nurse–Patient Relationship Scale; NPRS_PCN, patient‐centered nursing subscale of NPRS; ; NPRS_NPT, Nurse–Patient Trust subscale of NPRS; SD, standard deviation.

### 3.5. Mediation Links Between Study Variables

**FIGURE 1:**
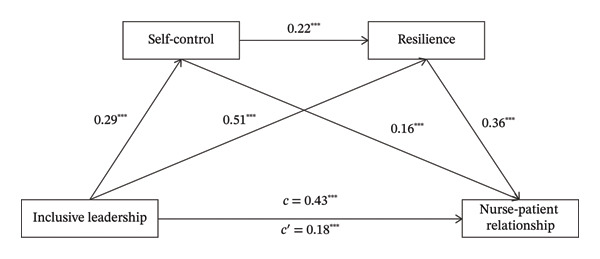
Model of the mediation role of self‐control and resilience in the relation between inclusive leadership and nurse–patient relationship. Standardized regression coefficients were displayed in the path diagram; *c*, total effect; *c*′, direct effect. Sex, age, educational years, income, and working years were treated as the covariates in the model. ^∗∗∗^, *p* < 0.001.

The results of the mediation analysis for the overall nurse‐perceived nurse–patient relationship quality are shown in Table 3 and illustrated in the path model (Figure 1). The total effect of inclusive leadership on the nurse–patient relationship quality was significant (*β* = 0.43, 95% CI: [0.35, 0.52]). When the mediators were included in the model, the direct effect remained significant but reduced in magnitude (*β* = 0.18, 95% CI: [0.08, 0.28]), supporting a model of partial mediation.

**TABLE 3:** Total, direct, and indirect effects of the mediation model.

Effect	*β*	Bootstrap SE	Bootstrap 95% CI
Total effect (Inclusive leadership ⟶ Nurse–patient relationship)	0.43	0.04	[0.35, 0.52]
Direct effect (Inclusive leadership ⟶ Nurse–patient relationship)	0.18	0.05	[0.08, 0.28]
Indirect effect	0.25	0.04	[0.18, 0.32]
Inclusive leadership ⟶ Self‐control ⟶ Nurse–patient relationship	0.05	0.01	[0.02, 0.07]
Inclusive leadership ⟶ Resilience ⟶ Nurse–patient relationship	0.18	0.03	[0.12, 0.25]
Inclusive leadership ⟶ Self‐control ⟶ Resilience ⟶ Nurse–patient relationship	0.02	0.01	[0.01, 0.04]

Abbreviations: CI, confidence interval; SE, standard error.

The total indirect effect through the proposed mediator chain was significant (*β* = 0.25, 95% CI: [0.18, 0.32]). Analysis of the specific pathways revealed: First, a significant independent indirect effect via self‐control: Inclusive leadership ⟶ Self‐control ⟶ Nurse‐patient relationship (*β* = 0.05, 95% CI: [0.02, 0.07]). Second, a significant independent indirect effect via resilience: Inclusive leadership ⟶ Resilience ⟶ Nurse‐patient relationship (*β* = 0.18, 95% CI: [0.12, 0.25]). Third, a significant sequential indirect effect: Inclusive leadership ⟶ Self‐control ⟶ Resilience ⟶ Nurse‐patient relationship (*β* = 0.02, 95% CI: [0.01, 0.04]). The full path coefficients (including B‐values, *β*‐values, standard errors, and *p* values) and *R* ^2^ values for each endogenous variable were present in Table S1. According to Cohen’s guidelines for interpreting effect size [50], the observed effects are small to medium, while the sequential indirect effect (*β* = 0.02) is small in magnitude. This modest sequential effect should be interpreted in the context of the complex, multidetermined nature of nurse–patient relationships, where multiple factors contribute incrementally to outcomes. Furthermore, parallel analyses conducted on the two subdimensions of the outcome variable yielded highly consistent findings. As detailed in Tables S2 and S3, both nurse–patient trust and patient‐centered nursing were significantly associated with inclusive leadership, with self‐control and resilience serving as significant parallel and sequential mediators. The corresponding path models are presented in Figures S1 and S2.

Finally, to address the potential influence of the CMB, the mediation analyses were rerun with the first latent factor from the CMB exploratory factor analysis included as an additional covariate. These analyses yielded results generally consistent with the primary findings. The indirect effect via self‐control (*β* = 0.03, 95% CI [0.01, 0.05]) and the indirect effect via resilience (*β* = 0.12, 95% CI [0.07, 0.17]) on the link of inclusive leadership with nurse‐perceived nurse–patient relationship quality remained significant. Furthermore, the sequential indirect effect of self‐control and resilience also remained significant (*β* = 0.01, 95% CI [0.00, 0.02]).

## 4. Discussion

This study sought to unravel the psychological mechanisms underlying the connection between inclusive leadership and the nurse‐perceived nurse–patient relationship quality by proposing and testing an integrated model with self‐control and resilience as parallel and sequential mediators. The findings provide support for our hypotheses, indicating that inclusive leadership is positively associated with nurse‐perceived nurse–patient relationship quality and that this link is significantly transmitted through both the independent and chained mediating roles of nurses’ self‐control and resilience. These results contribute to the literature by moving beyond establishing a direct association to delineating specific resource‐based pathways that may translate leadership climate into frontline relational care. It is important to emphasize that due to the cross‐sectional design, these findings represent associations rather than causal effects.

The significant direct association between inclusive leadership and the nurse‐perceived nurse–patient relationship quality is consistent with prior research on relational leadership models in healthcare [51]. This aligns with relational leadership theory, which emphasizes that leadership is a social process that shapes the quality of all ensuing interactions within a system [52]. An inclusive leader, by modeling respect, active listening, and responsiveness [5], may establish a normative blueprint for interpersonal conduct that nurses internalize and enact with patients. Furthermore, such leaders may make more equitable clinical assignments and buffer their teams from extraneous bureaucratic pressures, indirectly creating the temporal and cognitive space necessary for meaningful patient engagement [8]. The persistence of this direct effect, even after accounting for the mediators, suggests the existence of additional unexplored pathways, such as the leader’s role in shaping unit‐level psychological safety climate [9], which encourages open discussion of errors and patient concerns without fear of blame, thereby potentially improving collective learning and care coordination [53].

The confirmation of independent mediation via self‐control illuminates a critical self‐regulatory pathway consistent with COR theory [16]. The nursing environment is replete with potential “resource drains” (e.g., conflicting demands, emotional outbursts, and systemic inefficiencies) that may deplete the finite cognitive and emotional reserves necessary for self‐regulation [18]. Theoretically, an inclusive leader may alter this resource equation by acting as a “resource caravan passageway” [26], potentially reducing the key drains such as role ambiguity, unfair treatment, and supervisory stress. This conservation effect, if present, could protect self‐regulatory resources from workplace drains, which is crucial for maintaining job performance [54]. When nurses expend fewer volitional resources on managing a psychologically unsafe relationship with their supervisor or navigating a climate of perceived injustice, a greater reservoir of self‐control may be preserved for patient care. This available self‐control may allow nurses to pause before reacting, choose therapeutic communication strategies over defensive ones, and maintain professionalism in the face of provocation—behaviors foundational to building trust and demonstrating patient‐centeredness [3]. Research has consistently linked self‐control to better interpersonal functioning and conflict management [32, 55]. Therefore, the mediation via self‐control suggests that leadership quality may be associated not only with motivation but also with the professional discipline foundational to building patient trust.

Similarly, the independent mediation via resilience highlights a distinct capacity‐building pathway, aligning with social cognitive theory [21]. Resilience is not merely the absence of burnout but the presence of adaptive, recoverable strength. Inclusive leadership behaviors—such as actively soliciting input (openness), providing timely guidance (availability), and offering non‐judgmental support (accessibility)—may serve as sources of the key experiences that build this strength [56]. Theoretically, they may create mastery experiences by empowering nurses in decision‐making, foster vicarious learning by modeling adaptive coping strategies, and provide social persuasion through affirming feedback that reinforces a nurse’s belief in their own competence [57, 58]. When nurses perceive their leader as inclusive, they may internalize a sense of being valued and supported, which could strengthen their psychological capital [59]. This fortified resilience, in turn, may manifest in the nurse–patient relationship. A resilient nurse may possess the recovery agility to bounce back from a poor patient outcome, preventing one difficult interaction from negatively coloring the next [60]. Critically, resilience may sustain empathic concern by protecting against the compassion fatigue and depersonalization that can erode the core of patient‐centered care [61]. Overall, this pathway suggests that inclusive leadership may be associated with the long‐term adaptive capacity of the nursing workforce.

Perhaps the most notable finding of this study is the sequential mediation effect of self‐control and resilience. This finding offers a process‐oriented view of how personal resources may interact and accumulate, moving beyond static, independent effects. The data are consistent with the COR theory principle of “resource caravans,” where resources may coalesce and synergistically foster the development of other resources [26]. In this chain, self‐control acts as a foundational, gatekeeping resource. The successful daily regulation of attention, emotion, and behavior may prevent the gradual erosion and depletion of finite psychological resources, a process central to the development of burnout [62]. By consistently managing microstressors and emotional labor, a nurse may avoid a chronic state of resource loss that could undermine adaptive capacity [16]. This stable, well‐regulated baseline may create conditions in which resilience—a higher‐order, adaptive resource—can develop. A nurse who is not perpetually depleted may possess greater cognitive space and emotional energy to develop and utilize the positive coping strategies, cognitive reframing, and proactive seeking of support [20, 61]. However, it is important to acknowledge that alternative orderings (e.g., resilience preceding self‐control) are theoretically plausible, and the sequential pathway identified here is hypothesis‐driven and should not be read as evidence that self‐control temporally precedes resilience.

### 4.1. Limitations and Future Directions

This study has limitations that contextualize the findings and guide future research. First, the cross‐sectional design, while testing a theoretically driven model, cannot definitively establish causality or temporal ordering [63]. Mediation analysis in cross‐sectional data assumes a causal sequence that cannot be verified with this design. Longitudinal studies tracking these variables over multiple time points are needed to confirm the proposed temporal sequence and examine potential reciprocal effects (e.g., positive patient relationships may bolster a nurse’s resilience and self‐control). The possibility of reverse causality (e.g., nurses who report better patient relationships may perceive their leaders as more inclusive) cannot be ruled out.

Second, the study relies entirely on self‐reported data from a single source, including the outcome variable. We employed multiple diagnostic approaches to assess CMB: Harman’s single‐factor test, a CFA‐based common latent factor, and a statistical remedy for controlling analysis. While these analyses suggested that the CMB does not fully account for the observed associations, they do not eliminate the possibility of same‐source bias. Same‐source, self‐report designs may inflate observed associations due to response tendencies, social desirability bias, or transient mood states [42]. Future research would benefit from multisource and multimethod data, such as patient ratings of relationship quality, peer or supervisor assessments of self‐control and resilience behaviors, or objective indicators, to strengthen the validity of the findings.

Third, the sample was drawn from one cultural and geographic context (Sichuan Province, China). Cultural norms around leadership deference, power distance, emotional expression, and relationship‐building may influence the observed dynamics [64]. For example, in high power distance cultures, the effects of inclusive leadership may differ from those in more egalitarian cultural contexts. Replication in diverse cultural and healthcare system settings is essential to assess the model’s generalizability and boundary conditions. Furthermore, we did not collect department‐ or unit‐level identifiers, precluding formal examination of potential clustering effects. While our theoretical focus was on individual‐level psychological processes, unmeasured shared unit characteristics (e.g., unit leadership climate and workload norms) could theoretically influence the observed associations. Future research should explicitly model nested data structures by collecting unit identifiers and employing multilevel mediation frameworks.

Fourth, a particularly important limitation is the measurement of the nurse–patient relationship exclusively from the nurse’s perspective. While the NPRS is a validated instrument, it captures nurses’ perceptions of trust and patient‐centered care rather than the patient’s experience [38]. The patient perspective is central to the construct of therapeutic relationship quality. Future research should incorporate patient‐reported outcome measures of relationship quality, which may provide a more complete and less biased assessment.

Additionally, while statistically significant, some effects—particularly the sequential mediation effect (*β* = 0.02)—are small in magnitude. Small effects, while statistically significant in a large sample (*N* = 429), may have limited practical significance and should not be overinterpreted. Future research should examine whether this sequential pathway has clinically meaningful implications for patient outcomes or nurse well‐being. Finally, despite the inclusion of several covariates, other potential confounding variables may influence the observed associations. Personality traits (e.g., neuroticism and conscientiousness), which are known to correlate with self‐control, resilience, and work outcomes, were not measured. Unit‐level characteristics (e.g., patient acuity, nurse‐to‐patient ratio, and unit size) directly impact work demands that may also confound the relationships. Future studies should incorporate these variables into more comprehensive models to rule out alternative explanations [65].

### 4.2. Theoretical and Practical Implications

Theoretically, this study enriches the nursing leadership and work psychology literature in several ways. First, it integrates macrolevel leadership theory with microlevel psychological resource theories (i.e., COR and social cognitive theory) to elucidate a specific clinical outcome. Second, it identifies self‐control and resilience not merely as parallel outcomes of good leadership but as interlinked mediators in a serial chain, consistent with the resource caravan principle of COR theory [26]. Third, it shifts the focus of leadership outcomes in nursing beyond traditional metrics like turnover and burnout to a core clinical performance indicator—the nurse–patient relationship—thereby linking leadership literature directly with patient care quality [4, 66].

The practical implications of this study, while tempered by the cross‐sectional design, suggest potential directions for healthcare organizations. Organizations may consider cultivating inclusive leadership competencies through structured training in concrete skills such as active listening, soliciting input, and equitable decision‐making, supported by 360‐degree feedback tools for accountability [67]. Concurrently, interventions for staff that target both self‐control and resilience may be beneficial: evidence‐based techniques such as mindfulness‐based stress reduction may strengthen self‐control [68], while cognitive‐behavioral resilience training and peer mentoring may foster resilience [69]. The sequential mediation findings suggest a synergistic, tiered program design where foundational self‐regulation training should come first and may improve comprehensive resilience‐building [70]. Finally, leaders might consider workplace redesigns that reduce resource drains—such as streamlining documentation, ensuring fair workload distribution, and protecting time for nurse–patient interaction—to create an environment where sustaining self‐control and resilience is feasible [71].

## 5. Conclusion

In conclusion, this study provides initial evidence that inclusive leadership is associated with a stronger nurse‐perceived nurse–patient relationship quality, and this association is mediated by self‐control and resilience, both independently and sequentially. The findings suggest a pathway whereby supportive leadership may be associated with conserved and built personal resources, which may then be invested in the therapeutic work of patient care. This highlights that investing in the development of inclusive leaders and the psychological capital of the nursing workforce may be intrinsically linked strategies for achieving excellence in relational, patient‐centered care. Future research should adopt longitudinal, multimethod, and cross‐cultural approaches to further refine our understanding of these dynamic relationships.

## Funding

This work was supported by the Key Research and Development Program of Sichuan Province (Grant No. 2023YFS0084).

## Ethics Statement

Ethical approval for this study was secured from the Ethics Committee of West China Hospital of Sichuan University (Approval No.: 2021‐1216). All participants provided written informed consent, acknowledging the procedures and affirming their voluntary involvement.

## Conflicts of Interest

The authors declare no conflicts of interest.

## Supporting Information

Additional supporting information can be found online in the Supporting Information section.

## Supporting information


**Supporting Information** Table S1. Regression analysis of the relationship between variables. Table S2. Total, direct, and indirect effects of the mediation model for predicting nurse–patient trust. Table S3. Total, direct, and indirect effects of the mediation model for predicting patient‐centered nursing. Figure S1. Model of the mediation role of self‐control and resilience in the relation between inclusive leadership and nurse–patient trust. Figure S2. Model of the mediation role of self‐control and resilience in the relation between inclusive leadership and patient‐centered nursing.

## Data Availability

The data that support the findings of this study are available from the corresponding author upon reasonable request.
