# Supplementary material for: Inclusive Leadership Is Associated With Nurse‐Perceived Nurse‐Patient Relationship Quality Through the Mediation Link of Self‐Control and Resilience: A Cross‐Sectional Study
Source: J Nurs Manag. 2026 Jun 26;2026:7165226. doi: 10.1155/jonm/7165226 (PMC13305670; doi:10.1155/jonm/7165226)
Supplement: Supplementary file 1 — Supporting Information Table S1. Regression analysis of the relationship between variables. Table S2. Total, direct, and indirect effects of the mediation model for predicting nurse–patient trust. Table S3. Total, direct, and indirect effects of the mediation model for predicting patient‐centered nursing. Figure S1. Model of the mediation role of self‐control and resilience in the relation between inclusive leadership and nurse–patient trust. Figure S2. Model of the mediation role of self‐control and resilience in the relation between inclusive leadership and patient‐centered nursing. [file JONM-2026-7165226-s001.docx]

**Supplementary Material for**

***‘Inclusive leadership is associated with nurse-perceived nurse-patient relationship quality through the mediation link of self-control and resilience: A cross-sectional study’***

**Table S1.** Regression analysis of the relationship between variables

**Table S2.** Total, direct, and indirect effects of the mediation model for predicting nurse-patient trust

**Table S3.** Total, direct, and indirect effects of the mediation model for predicting patient-centered nursing

**Figure S1.** Model of the mediation role of self-control and resilience in the relation between inclusive leadership and nurse-patient trust

**Figure S2.** Model of the mediation role of self-control and resilience in the relation between inclusive leadership and patient-centered nursing

**Table S1.** Regression analysis of the relationship between variables

| **Model** | **Outcome variable** | **Predictor variable** | **B** | **SE** | **β** | **t** | **R²** |
| --- | --- | --- | --- | --- | --- | --- | --- |
| Model 1 | Nurse-patient relationship | Inclusive leadership | 0.42 | 0.04 | 0.43 | 9.92^***^ | 0.21 |
| Model 2 | Self-control | Inclusive leadership | 0.38 | 0.06 | 0.29 | 6.15^***^ | 0.11 |
| Model 3 | Resilience | Inclusive leadership | 0.55 | 0.04 | 0.51 | 12.69^***^ | 0.38 |
|  |  | Self-control | 0.17 | 0.03 | 0.22 | 5.38^***^ |  |
| Model 4 | Nurse-patient relationship | Inclusive leadership | 0.18 | 0.05 | 0.18 | 3.71^***^ | 0.34 |
|  |  | Self-control | 0.11 | 0.03 | 0.16 | 3.65^***^ |  |
|  |  | Resilience | 0.33 | 0.05 | 0.36 | 7.10^***^ |  |

Note. SE, standard error; ^***^, p < 0.001.

**Table S2.** Total, direct, and indirect effects of the mediation model for predicting nurse-patient trust

| Effect | Effect size | Bootstrap SE | Bootstrap 95% CI |
| --- | --- | --- | --- |
| Total effect (Inclusive leadership → Nurse-patient trust) | 0.40 | 0.04 | [0.31, 0.49] |
| Direct effect (Inclusive leadership → Nurse-patient trust) | 0.14 | 0.05 | [0.04, 0.24] |
| Indirect effect | 0.26 | 0.04 | [0.19, 0.34] |
| Inclusive leadership → Self-control → Nurse-patient trust | 0.04 | 0.01 | [0.02, 0.07] |
| Inclusive leadership → Resilience → Nurse-patient trust | 0.20 | 0.03 | [0.13, 0.27] |
| Inclusive leadership → Self-control → Resilience → Nurse-patient trust | 0.02 | 0.01 | [0.01, 0.04] |

*Note.* SE, standard error; CI, Confidence Interval.

**Table S3.** Total, direct, and indirect effects of the mediation model for predicting patient-centered nursing

| Effect | Effect size | Bootstrap SE | Bootstrap 95% CI |
| --- | --- | --- | --- |
| Total effect (Inclusive leadership → Patient-centered nursing) | 0.43 | 0.04 | [0.34, 0.51] |
| Direct effect (Inclusive leadership → Patient-centered nursing) | 0.20 | 0.05 | [0.11, 0.30] |
| Indirect effect | 0.23 | 0.04 | [0.16, 0.30] |
| Inclusive leadership → Self-control → Patient-centered nursing | 0.05 | 0.01 | [0.02, 0.07] |
| Inclusive leadership → Resilience → Patient-centered nursing | 0.16 | 0.03 | [0.10, 0.22] |
| Inclusive leadership → Self-control → Resilience → Patient-centered nursing | 0.02 | 0.01 | [0.01, 0.03] |

*Note.* SE, standard error; CI, Confidence Interval.





**Figure S1.** Model of the mediation role of self-control and resilience in the relation between inclusive leadership and nurse-patient trust. Standardized regression coefficients were displayed in the path diagram; c, total effect; c’, direct effect. Sex, age, educational years, income, and working years were treated as the covariates in the model. ^***^, p < 0.001; ^**^, p < 0.01.





**Figure S2.** Model of the mediation role of self-control and resilience in the relation between inclusive leadership and patient-centered nursing. Standardized regression coefficients were displayed in the path diagram; c, total effect; c’, direct effect. Sex, age, educational years, income, and working years were treated as the covariates in the model. ^***^, p < 0.001.
